# Exploration of Shared Gene Signatures and Molecular Mechanisms Between Periodontitis and Nonalcoholic Fatty Liver Disease

**DOI:** 10.3389/fgene.2022.939751

**Published:** 2022-06-28

**Authors:** Wanqiu Xu, Zhengwei Zhang, Lihong Yao, Bing Xue, Hualei Xi, Xiumei Wang, Shibo Sun

**Affiliations:** ^1^ Department of Dentistry, The Second Affiliated Hospital of Harbin Medical University, Harbin, China; ^2^ Ward 7, Department of General Surgery, The Second Affiliated Hospital of Harbin Medical University, Harbin, China

**Keywords:** periodontitis, nonalcoholic fatty liver disease, WGCNA, dendritic cell migration, miRNAs–mRNAs

## Abstract

**Background:** Periodontitis is associated with periodontal tissue damage and teeth loss. Nonalcoholic fatty liver disease (NAFLD) has an intimate relationship with periodontitis. Nevertheless, interacted mechanisms between them have not been clear. This study was intended for the exploration of shared gene signatures and latent therapeutic targets in periodontitis and NAFLD.

**Methods:** Microarray datasets of periodontitis and NAFLD were obtained from the Gene Expression Omnibus (GEO) database. The weighted gene co-expression network analysis (WGCNA) was utilized for the acquisition of modules bound up with NAFLD and periodontitis. We used ClueGO to carry out biological analysis on shared genes to search their latent effects in NAFLD and periodontitis. Another cohort composed of differential gene analysis verified the results. The common microRNAs (miRNAs) in NAFLD and periodontitis were acquired in the light of the Human microRNA Disease Database (HMDD). According to miRTarbase, miRDB, and Targetscan databases, latent target genes of miRNAs were forecasted. Finally, the miRNAs–mRNAs network was designed.

**Results:** Significant modules with periodontitis and NAFLD were obtained *via* WGCNA. GO enrichment analysis with GlueGo indicated that damaged migration of dendritic cells (DCs) might be a common pathophysiologic feature of NAFLD and periodontitis. In addition, we revealed common genes in NAFLD and periodontitis, including IGK, IGLJ3, IGHM, MME, SELL, ENPP2, VCAN, LCP1, IGHD, FCGR2C, ALOX5AP, IGJ, MMP9, FABP4, IL32, HBB, FMO1, ALPK2, PLA2G7, MNDA, HLA-DRA, and SLC16A7. The results of differential analysis in another cohort were highly accordant with the findings of WGCNA. We established a comorbidity model to explain the underlying mechanism of NAFLD secondary to periodontitis. Finally, the analysis of miRNA pointed out that hsa-mir-125b-5p, hsa-mir-17-5p, and hsa-mir-21-5p might provide potential therapeutic targets.

**Conclusion:** Our study initially established a comorbidity model to explain the underlying mechanism of NAFLD secondary to periodontitis, found that damaged migration of DCs might be a common pathophysiological feature of NAFLD and periodontitis, and provided potential therapeutic targets.

## Introduction

Nonalcoholic fatty liver disease (NAFLD), accompanied by varying levels of hepatic fat accumulation, can gradually progress to nonalcoholic steatohepatitis, cirrhosis, and hepatocellular carcinoma, which has fatal consequences (Wesolowski et al., 2017). It was reported that the prevalence of NAFLD accounted roughly 25%, with the prospect of further increase according to expanding populations with metabolic syndrome (Younossi et al., 2016; Estes et al., 2018). According to the pathophysiology of NAFLD, some kinds of medical treatments with respective effects are being assessed in clinical trials. It is regrettable that these drug candidates have been found bringing unpalatable side effects or are limited by efficacy (Alkhouri et al., 2020; Younossi et al., 2021; Barritt et al., 2022). Currently, there has been a lively investigation over the participation of periodontitis in the occurrence and development of NAFLD. Some scholars even believed that there was a comorbidity effect between the two diseases (Rosato et al., 2019). Suffering from oral microbial imbalance brought about by anaerobic Gram‐negative bacteria chiefly, periodontitis is associated with periodontal tissue damage and teeth loss (Kuraji et al., 2021). It was reported that there were 1.1 billion people with severe periodontitis worldwide in 2019 (Chen et al., 2021). Actually, mechanical debridement is hard to absolutely clear periodontitis infection and prolonged antibiotic exposure is effective but unsafe (Rotundo et al., 2010; Rams et al., 2020).

From the beginning, periodontitis has contributed to the development of NAFLD owing to systemic inflammation and oxidative stress on the basis of vitro study (Tomofuji et al., 2007). Then, Porphyromonas gingivalis, the main pathogenic bacteria of periodontitis, resulted in the development of NAFLD, above which academic discussion had continued ever since (Furusho et al., 2013; Nagasaki et al., 2021; Yamazaki et al., 2021). Epidemiological investigation reported that NAFLD incidence was increasing with the combination of periodontitis, which could increase the risk of progression to liver fibrosis as well (Akinkugbe et al., 2017a; Akinkugbe et al., 2017b; Iwasaki et al., 2018; Suominen et al., 2019; Kuroe et al., 2021). The potential associations between periodontitis and NAFLD has been discussed from *in vitro*, *in vivo*, and epidemiologic perspectives, but the genetic and biological mechanisms of connection between periodontitis and NAFLD is unknown. Although most studies suggest that periodontitis can affect NAFLD outcomes, the effect of genetic and biological mechanisms might be bidirectional and extremely valuable.

In order to have insights into the mechanisms of diseases, gene microarray technology is developed, which can generate thousands of gene expression data in various diseases. Despite periodontitis and NAFLD being two relatively independent pathological process, periodontitis feels more like a trigger, once it is lit, it will quicken NAFLD aggravation. To explain the trigger, the weighted gene co-expression network analysis (WGCNA) was applied to seek the clusters of shared genes in periodontitis and NAFLD. This method has been utilized to explain genetic mechanism related to various disease phenotypes effectively (Zhu et al., 2020; Yao et al., 2021). Through the deep analysis of the Gene Expression Omnibus (GEO) database, we found that genes related to “dendritic cell migration” were presented in modules hugely relevant to periodontitis and NAFLD, which meant that biological pathway “dendritic cell migration” might play a significant role in periodontitis and NAFLD. In addition, the unique gene signatures in periodontitis and NAFLD were also identified and microRNAs (miRNAs) might play a regulatory role. So far as we know, this is the first study to utilize the bioinformation technique to explain the gene signatures between periodontitis and NAFLD, which is expected to provide new diagnostic and therapeutic windows for these two diseases.

## Methods

### Download and Preprocessing of the Gene Expression Omnibus Dataset

We used the key words “Nonalcoholic Fatty Liver Disease” or “periodontitis” to search NAFLD and periodontitis gene expression profiles in which the data at original or processed state could be for the return to analysis in the GEO database (Barrett et al., 2013). Finally, the GEO dataset numbered GSE16134 was accepted, which contained a total of 241 periodontitis samples and 69 healthy samples. The GSE48452 and GSE63067 microarray datasets were used for NAFLD, which contained raw transcriptomics data from the human liver tissue. In GSE48452 dataset, 73 samples of human liver grouped into C (control = 14), H (healthy obesity = 27), S (steatosis = 14), and N (NASH = 18) from original references. In GSE63067dataset, two human steatosis and nine human nonalcoholic steatohepatitis (NASH) together with their respective control patterns were analyzed from original references. The original data were processed with background correction, normalization, and relative expression calculation. Log2 transformation was applied to gene expression profiling and the probes were matched with their gene symbols on the basis of annotated files from relevant platforms. Ultimately, we acquired the genetic matrix with row and column defined as specimen names and gene symbols, respectively, for the following analysis.

### Weighted Gene Co-Expression Network Analysis

A popular algorithm, WGCNA, is applied to seek gene co-expression modules with the great importance of biology and discover the relevance between diseases and gene networks (Langfelder and Horvath, 2008). Consequently, WGCNA was utilized for the acquisition of modules bound up with NAFLD and periodontitis. All the differential genes (DEGs) from healthy and disease samples satisfying *p* value < 0.05 were collected for WGCNA analysis (Supplementary Data S1). Clustering of samples was doing well and the threshold of cutting line was 30. The soft thresholds ranging from 1 to 20 were used for topology calculation and optimum soft threshold was identified as 6. According to the soft threshold, the matrix of correlations was converted to the adjacency matrix and then into a topological overlap matrix (TOM). With the average-linkage hierarchical clustering method which followed, the genes were clustered. The modules were divided according to TOM, each of which contained at least 50 genes. The cutting height of gene module was 0.7 and similar modules were combined. After that, gene significance (GS) and module membership (MM) in every module were calculated for plotting the scatter plots. At last we applied Pearson correlation analysis to estimate the relevance of disease emergence with the merged modules.

### Identification of Shared and Unique Gene Signatures

The modules with high correlation with NAFLD and periodontitis were chosen and the shared genes in modules positively related to NAFLD and periodontitis were crossed and overlapped through venn (Bardou et al., 2014). The nonredundant GO terms can be classified and visually arranged into networks grouped by functions through ClueGO, which is a Cytoscape plug-in unit (Bindea et al., 2009). Hence, we used ClueGO to carry out biological analysis on the shared genes to search their latent effects in NAFLD and periodontitis, in which the biological process (BP) of GO analysis was highlighted. The unique gene signatures in NAFLD and periodontitis were distinguished through the protein–protein interaction (PPI) network and cluster analysis, the latter of which was calculated by the “MCODE” algorithm with default parameters in Cytoscape software (version: 3.7.2).

## Results

### The Co-Expression Modules in Periodontitis and Nonalcoholic Fatty Liver Disease

**FIGURE 1:**
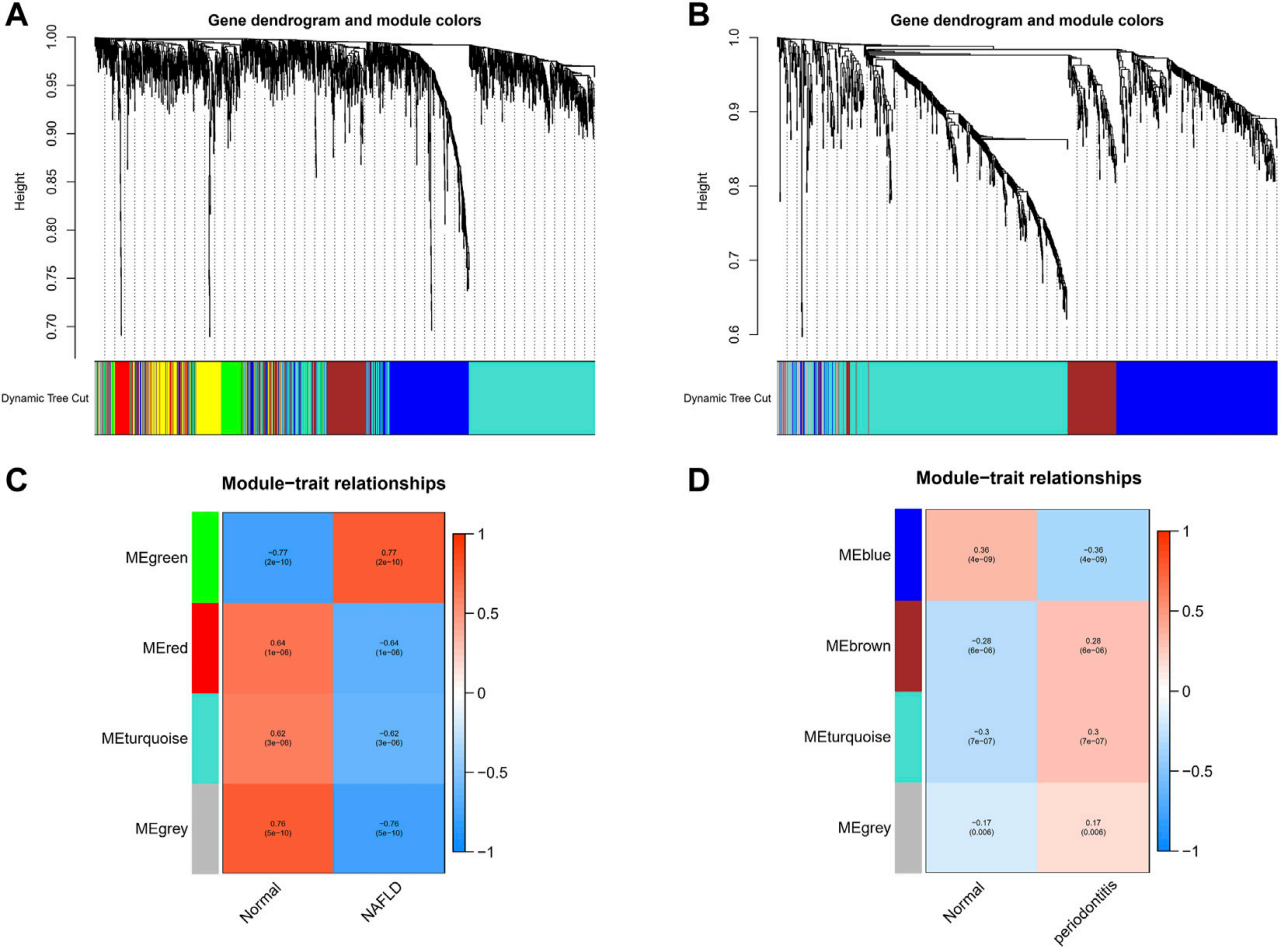
Weighted gene co-expression network analysis (WGCNA). **(A)** The cluster dendrogram of co-expression genes in NAFLD. **(B)** The cluster dendrogram of co-expression genes in periodontitis. **(C)** Module–trait relationships in NAFLD. Each cell contains the corresponding correlation and *p*-value. **(D)** Module–trait relationships in periodontitis. Each cell contains the corresponding correlation and *p*-value. NAFLD, nonalcoholic fatty liver disease.

With the application of WGCNA, four modules in total were recognized in GSE48452 and GSE63067, each of which had different color betokening separate module. For the assessment of relevance between disease and each module, a heatmap was plotted on the basis of Spearman correlation coefficient, in which module “green” had the highest relevance to NAFLD (Figures 1A,C). The module, with the core (r = 0.77), was positively related to NAFLD, including 920 genes. Four modules in total were recognized equally in GSE16134, in which the module “cyan” was the strongest and positively related to periodontitis (r = 0.3), including 522 genes (Figures 1B,D).

### The Common Gene Signatures in Periodontitis and Nonalcoholic Fatty Liver Disease

**FIGURE 2:**
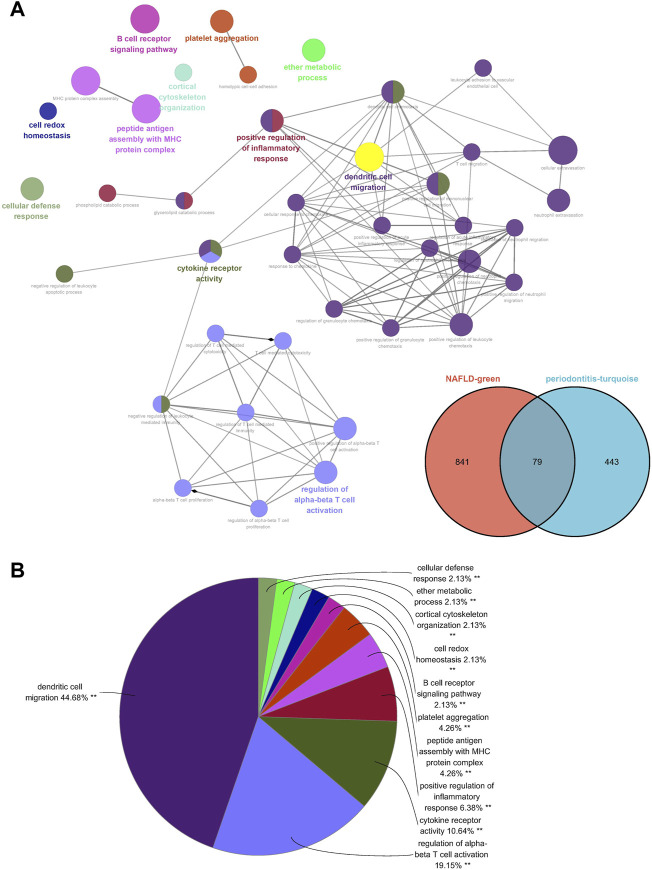
ClueGO enrichment analysis. **(A)** The interaction network of GO terms generated by the Cytoscape plug-in ClueGO. **(B)** Proportion of each GO terms group in the total. GO, gene ontology. **p < 0.05.

Seventy-nine genes were crossed and overlapped in the relevant core modules of NAFLD and periodontitis, which was recognized as gene set 1 (GS1). Periodontitis could be the important risk factor for the development of NAFLD according to current study. GlueGo was used to discuss the latent functions of GS1 through the GO enrichment analysis. The top three markedly enriched GO terms about BP were “dendritic cell migration,” “regulation of alpha-beta T cell activation,” and “cytokine receptor activity” (Figure 2A). Dendritic cell migration represented 44.68% of all the GO terms (Figure 2B), meaning that this pathway might be vital to both NAFLD and periodontitis.

### The Unique Gene Signatures in Periodontitis and Nonalcoholic Fatty Liver Disease

**FIGURE 3:**
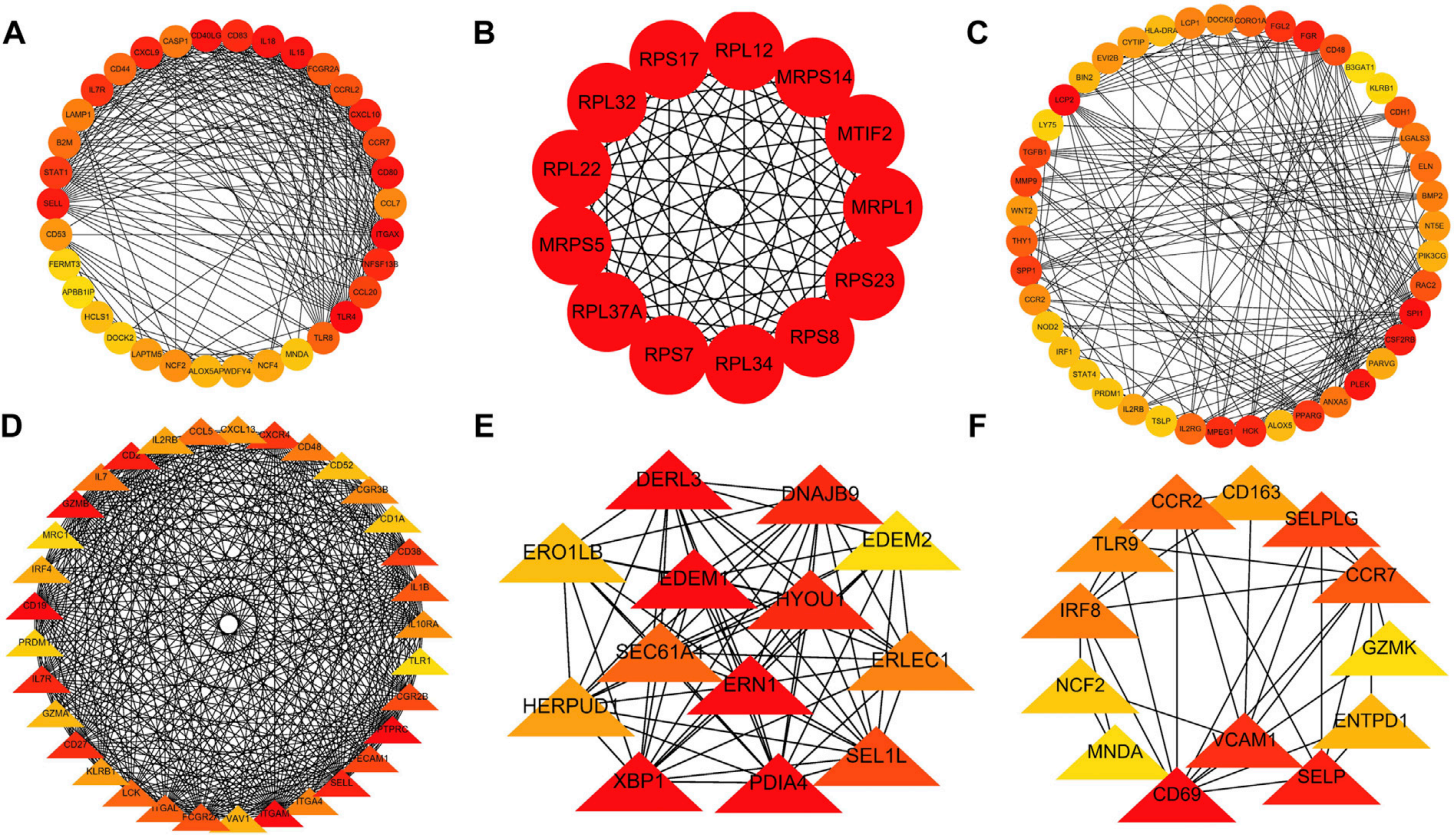
The PPI network. **(A–C)** The clusters 1-3 extracted from green module in NAFLD. **(D–F)** The clusters 1-3 extracted from the cyan module in periodontitis. PPI, protein–protein network; NAFLD, nonalcoholic fatty liver disease.

**FIGURE 4:**
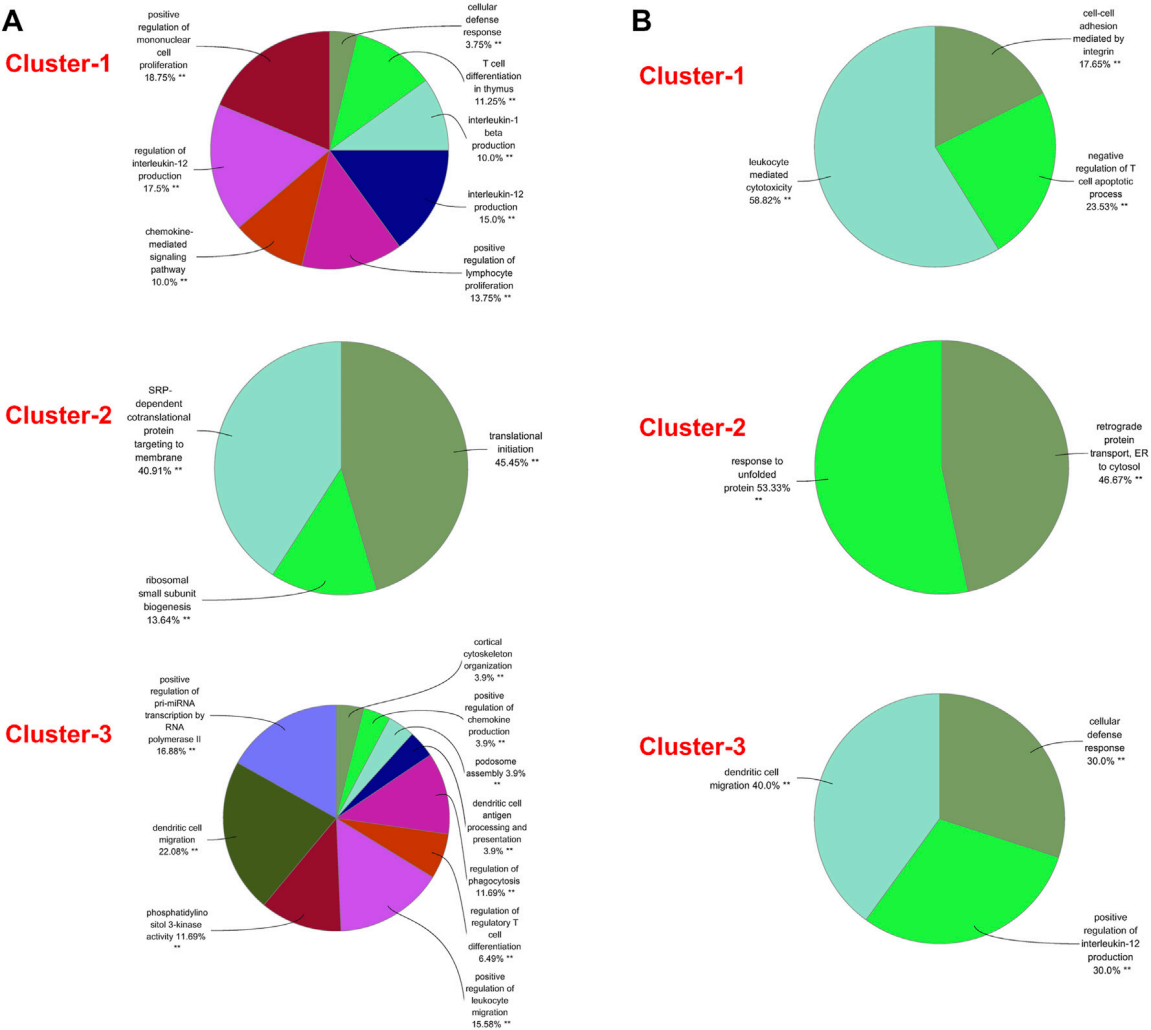
GO biological process analyses of clusters. **(A)** The GO biological process analyses of three gene clusters in NAFLD. **(B)** The GO biological process analyses of three genes clusters in periodontitis. GO, gene ontology; NAFLD, nonalcoholic fatty liver disease. **p < 0.05.

A PPI network was subsequently established at protein levels for green module of NAFLD. MCODE analysis was applied to acquire clusters. There were 34 nodes and 274 edges in cluster 1 (score = 16.606) (Figure 3A). Cluster 2 embodied 13 nodes and 78 edges (score = 13.000) (Figure 3B). Cluster 3 embodied 43 nodes and 209 edges (score = 9.952) (Figure 3C). Cluster 3 was primarily related to dendritic cell migration, which was represented with the functional enrichment analysis (Figure 4A). Consequently, it was inferred that cluster 3 pertained to common genes section from NAFLD and periodontitis. The other two clusters were recognized as unique gene signatures in NAFLD. The PPI network was established at protein levels for cyan module of periodontitis equally. MCODE analysis was also applied to acquire the clusters. There were 33 nodes and 387 edges in cluster 1 (score = 24.188) (Figure 3D). Cluster 2 embodied 13 nodes and 71 edges (score = 11.833) (Figure 3E). Cluster 3 embodied 13 nodes and 34 edges (score = 5.667) (Figure 3F). Coincidentally, cluster 3 was primarily related to dendritic cell migration, which was represented with the functional enrichment analysis (Figure 4B). The other two clusters were recognized as unique gene signatures in periodontitis.

### The Differential Genes Analysis in Periodontitis and Nonalcoholic Fatty Liver Disease

**FIGURE 5:**
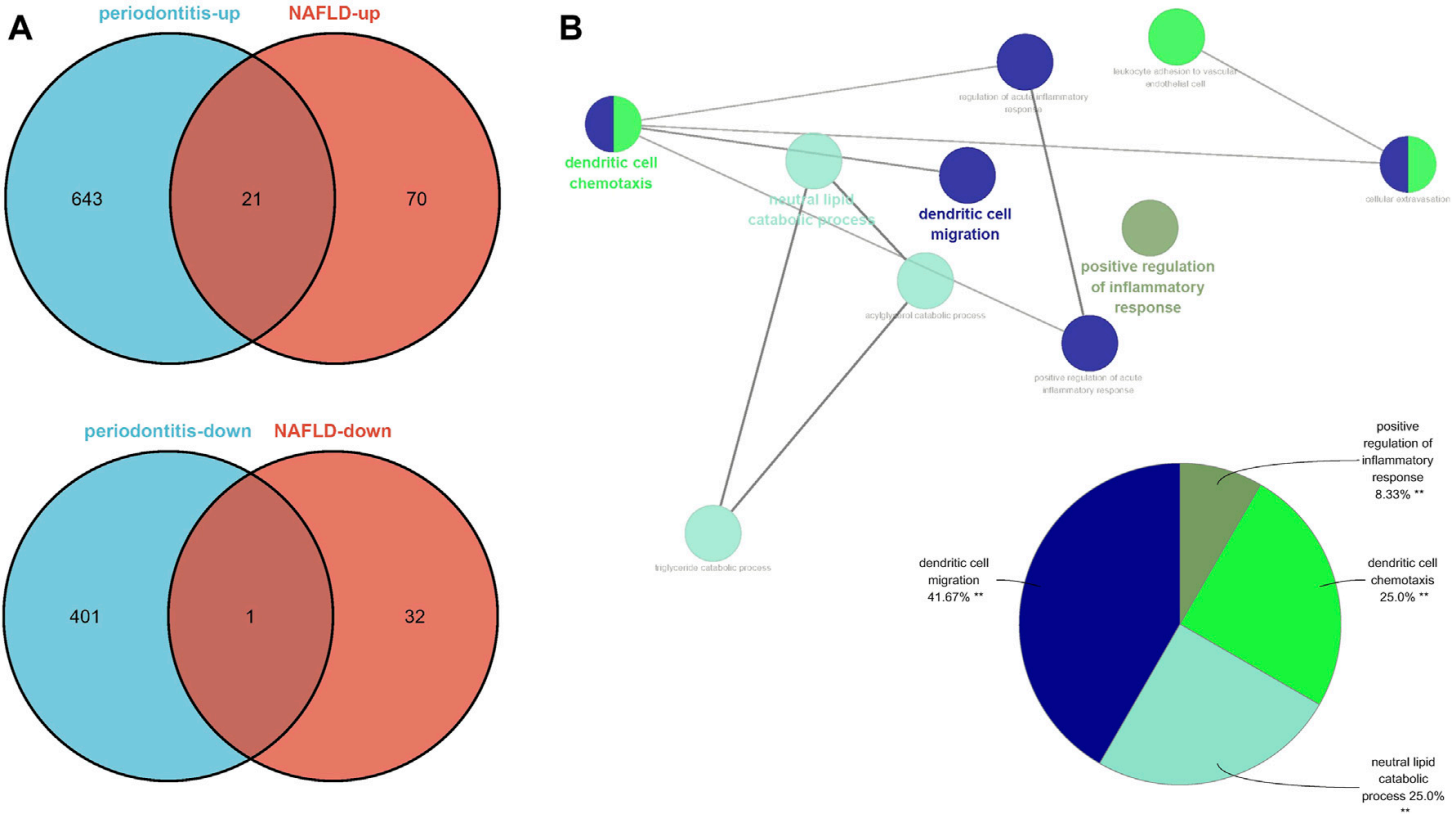
Identification of the common DEGs and ClueGO enrichment analysis. **(A)** The Venn diagram of the upregulated and downregulated genes in periodontitis and NAFLD. **(B)** The interaction network of GO terms generated by the Cytoscape plug-in ClueGO and proportion of GO terms in the total. GO, gene ontology; NAFLD, nonalcoholic fatty liver disease. **p < 0.05.

There were 91 upregulated genes and 33 downregulated genes being represented in GSE48452 and GSE63067. Concurrently, there were 664 upregulated genes and 402 downregulated genes represented in GSE16134. Of all the upegulated genes, 21 overlapped genes were discovered (IGK, IGLJ3, IGHM, MME, SELL, ENPP2, VCAN, LCP1, IGHD, FCGR2C, ALOX5AP, IGJ, MMP9, FABP4, IL32, HBB, FMO1, ALPK2, PLA2G7, MNDA, and HLA-DRA). On the other hand, one downregulated gene was overlapped (SLC16A7), which were recognized as gene set 2 (GS2) (Figure 5A). Representing remarkable enrichment of dendritic cell migration, dendritic cell chemotaxis, and neutral lipid catabolic process, the genes of GS2 were explored through the functional enrichment analysis, which was highly accordant with the findings of WGCNA (Figure 5B).

### Identification and Analysis of Common miRNAs in Periodontitis and Nonalcoholic Fatty Liver Disease

**FIGURE 6:**
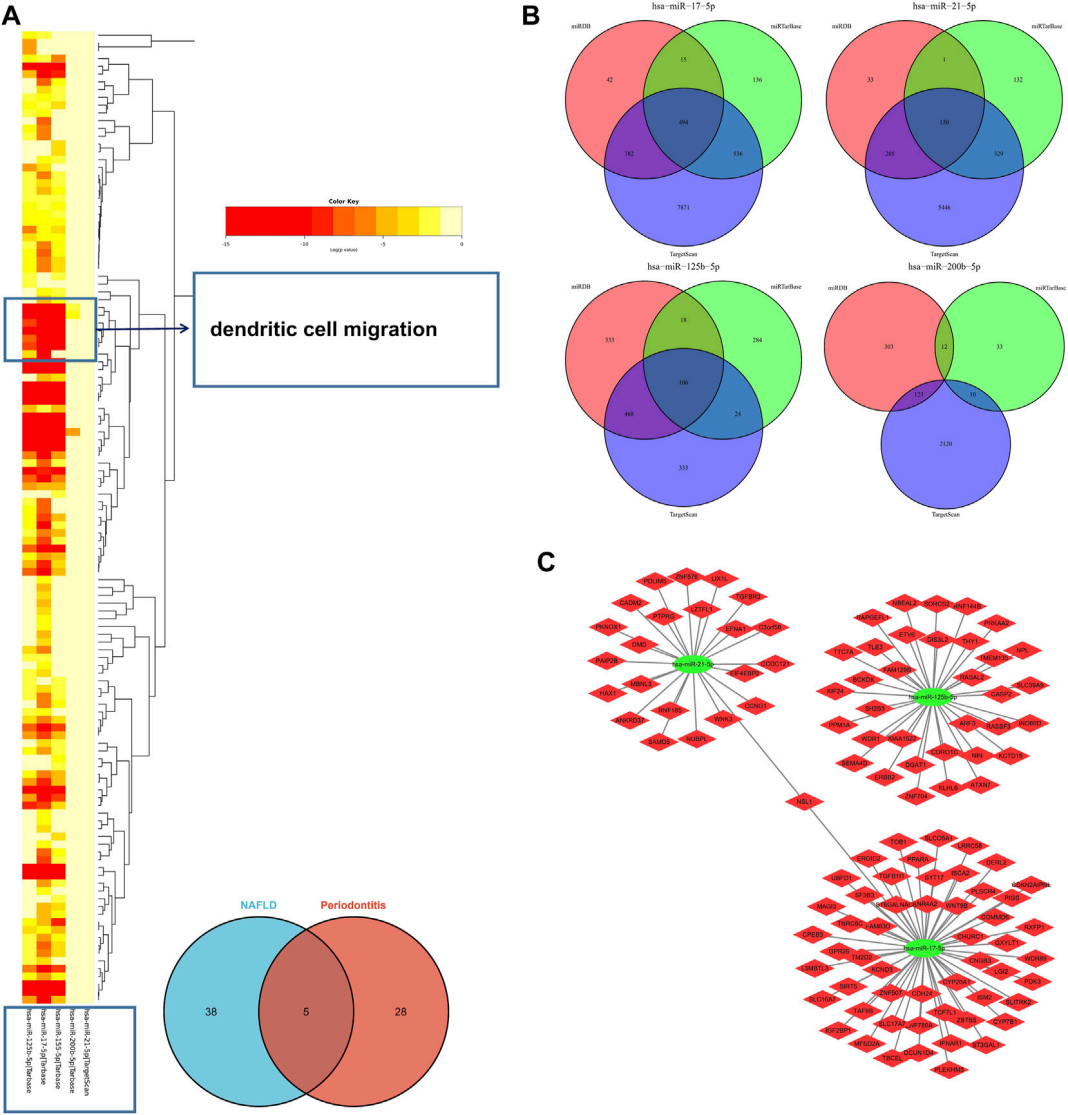
**(A)** The functional enrichment analysis of five common miRNAs. The arrow indicated the dendritic cell migration signaling pathway. **(B)** The Venn diagram of predicted target genes of miRNAs according to miRTarbase, miRDB, and Targetscan databases. **(C)** MiRNAs–mRNAs network. MiRNAs, microRNAs.

In the light of the Human microRNA Disease Database (HMDD) (Huang et al., 2019), 43 miRNAs were found to be related to NAFLD and 33 miRNAs were related to periodontitis (Supplementary Data S2). There were five overlapped miRNAs (hsa-mir-125b-5p, hsa-mir-155-5p, hsa-mir-17-5p, hsa-mir-200b-5p, and hsa-mir-21-5p) between NAFLD and periodontitis. There followed the enrichment analysis of five miRNAs, which revealed a variety of biological functions that these miRNAs are involved in. Similarly, “dendritic cell migration” got involved in these biological processes according to the heatmap, signifying that miRNAs associated with pathogenesis of NAFLD and periodontitis could also regulate dendritic cell migration (Figure 6A). Hence, our findings were proved again. According to miRTarbase (Chou et al., 2018), miRDB (Chen and Wang, 2020), and Targetscan (Morovat et al., 2022) databases, latent target genes of five miRNAs were forecasted (Figure 6B). Unfortunately, hsa-mir-155-5p was not retrieved in the database, and hsa-mir-200b-5p had no overlapped target genes. Finally, the miRNAs–mRNAs network was designed (Figure 6C).

## Discussion

As noted earlier, NAFLD has a high prevalence in periodontitis, indicating that some susceptibility factors in periodontitis may trigger the initiation and progression of NAFLD. Although it is not yet clear that how hazardous factors are delivered to liver from periodontium, the following two routes have been highly accepted. Blood transmission of bacteria, endotoxin, and inflammatory mediators from the periodontal tissues is the first aspect correlating periodontitis and NAFLD. Delivery of oral bacteria *via* the digestive tract is the second aspect, which brings out the imbalance of the intestinal bacteria (Kuraji et al., 2021). Regardless of dangerous medium such as periodontal bacteria, lipopolysaccharide and proinflammatory mediators, or intestinal dysbacteriosis, the precise role of them in effect of periodontitis on NAFLD needs further studies. So far, no studies have discussed the susceptibility of NAFLD in periodontitis at the genetic level.

Drawing support from WGCNA, we first discussed the common mechanisms of periodontitis and NAFLD. The differentially expressed genes in common were found in the intersection of GS1 and GS2, such as VCAN, LCP1, and ENPP2. Functional enrichment analysis concerned included dendritic cell migration, regulation of alpha-beta T cell activation, cytokine receptor activity, dendritic cell chemotaxis, and neutral lipid catabolic process. Finally, the miRNAs–mRNAs network was designed. More importantly, genes related to “dendritic cell migration” were presented in modules hugely relevant to periodontitis and NAFLD and experienced repeated verification. In addition, miRNAs might play a regulatory role in periodontitis and NAFLD. Playing a major role in innate immunity, dendritic cells (DCs) could capture and present antigens, which are also the bond to adaptive immunity (Steinman, 2001). The research shows that transmission of bacteria from periodontal tissues to distant sites *via* systemic circulation might appear at highly migrated DCs (Carrion et al., 2012). Porphyromonas gingivalis, as major pathogens in periodontitis, can attack DCs, reduce the level of proapoptosis protein expression, and prolong the survival of DCs (Meghil et al., 2019). This type of bacteria not only damages immune homeostasis of DCs, but also disrupts DCs homing to secondary lymphoid organs, the latter of which makes the inflammation migrate to vascular circulation (Miles et al., 2014). Unfortunately, it could avoid intracellular killing in DCs by targeting to dendritic cell-specific intercellular adhesion molecule-3-grabbing nonintegrin (El-Awady et al., 2015). However, oral microbial diversity destines that Porphyromonas gingivalis do not fight alone. In a previous study, a union of three oral microorganisms, *Streptococcus gordonii, Fusobacterium nucleatum, and Porphyromonas gingivalis*, drove bacterial growth, attack and stability in DCs, and regressed DCs maturation *via* coordinated effects, which generated microbial transmission and inflammatory spread (El-Awady et al., 2019). After-effects of bacteria themselves are taken out, lipopolysaccharide or proinflammatory cytokines, coming from periodontitis and bringing about low‐grade systemic inflammatory state, is closely related to DCs (Kanaya et al., 2004; Jardine et al., 2019; Psarras et al., 2021). With its receptors distributed extensively in the human body, inactive gingipains, as critical virulence factors of *Porphyromonas gingivalis*, leads to proinflammatory response in DCs (Ciaston et al., 2022). All in all, DCs not only play a central role in initiating and exacerbating periodontitis but also could be considered as potential contributing factors to the development of systemic diseases related to periodontitis, one of which is NAFLD.

It is generally known that intestinal microbial imbalance is intimately connected to NAFLD. First, anomalous abundance changes of bacterial phyla affect the severity of NAFLD (Boursier et al., 2016). Second, metabolite of intestinal bacteria results in fatty degeneration of liver cells, insulin resistance, and hepatic fibrosis (Ji et al., 2019). Third, endotoxemia attributed to the increase in intestinal permeability is related to pathogenesis of NAFLD (Wang et al., 2022). However, the mechanism of the pathology in which the intestinal flora imbalance induced by oral bacteria contributes to NAFLD has been unclear. Studies have pointed out that *Porphyromonas gingivalis* plays a major role in the process *via* interfering with the metabolic and immune profiles (Wang et al., 2022). It is not clear if DCs also affect the transmission of pathogenic bacteria and their toxic metabolites to the liver through the portal vein. The existing fact remains that the physiological action of DCs can be affected by the intestinal microbes (Yang et al., 2021). On the other hand, numerous researches have proved that migratory DCs could dominate induction of enteric T regulatory cells to manage commensal bacteria or to set up oral tolerance targeted at dietary antigens (Esterházy et al., 2016; Esterházy et al., 2019; Russler-Germain et al., 2021). Although the action mechanism of DCs in NAFLD is not completely clear, existing studies have confirmed the important role of DCs. DCs play a proinflammatory role in the animal models with nonalcoholic steatohepatitis (NASH). With CD11c+ DCs or CD103+ DCs consumption, decreased expression of proinflammatory cytokines and chemokines could prevent liver fibrosis (Nati et al., 2016; Schuster et al., 2018). Recent research has also shown that depletion of type 1 conventional DCs attenuates liver pathology in the NASH mouse models (Deczkowska et al., 2021). As noted previously, NAFLD has a high morbidity in periodontitis, indicating that predisposing factors in periodontitis could touch off NAFLD. In our modeling, both the discovery cohort and validation cohort reached the conclusion that dendritic cell migration played an important part in gene function enrichment analysis. Previous studies also support our view. Consequently, damaged migration of DCs might be a common pathophysiologic feature of NAFLD and periodontitis, which means that dendritic cell migration plays a key role and provides critical therapeutic target in the comorbidity model. MiRNA, as endogenous noncoding regulatory RNA, plays huge roles in the regulation of post-transcriptional gene. We have constructed the miRNAs–mRNAs network with the benefit of HMDD, miRTarbase, miRDB, and Targetscan databases. Interestingly, the target genes of common miRNAs, having no intersection with GS1 and GS2, still enriched in “dendritic cell migration”, which might be related to the indirect interaction of genes. Among these miRNAs, epigenetic silencing of miR-125b-5p resulted in liver fibrosis in NAFLD (Cai et al., 2020). Differential expression of miR-125b-5p influenced the functions of DCs (Hu et al., 2017). Mast cells had a close associate of periodontitis, and overexpressed miR-125b-5p in its own exosomes (Ekström et al., 2012; Tetè et al., 2021). We speculated that periodontitis might be affected by miR-125b-5p. Similarly, miR-17-5p and miR-21-5p were reported to play a part in periodontitis and migration of DCs and were predicted to get involved in NAFLD (Du et al., 2016; Kim et al., 2017; Reis et al., 2018; Cui et al., 2019; Zhang et al., 2020; Lin et al., 2022). Although these miRNAs have not been verified in the microenvironment of comorbidity with periodontitis and NAFLD, they also provide important therapeutic targets.

Considering the reality of the situation, experimental validation is currently not possible because clinical specimens of NAFLD are extremely difficult to obtain. Therefore, this is a limitation of our study and we will gradually collect samples for vitro assays. All in all, our study has established a comorbidity model to explain the underlying mechanism of NAFLD secondary to periodontitis, found that damaged migration of DCs might be a common pathophysiologic feature of NAFLD and periodontitis, and provided potential therapeutic targets.

## Data Availability

The original contributions presented in the study are included in the article/Supplementary Material. Further inquiries can be directed to the corresponding authors.

## Supplementary Material

Supplementary Data S1 All differential genes (DEGs) from health and disease samples.

Supplementary Data S2 43 miRNAs related to NAFLD and 33 miRNAs related to periodontitis according to HMDD. HMDD, Human microRNA Disease Database.
